# Amyloid beta induces *Fmr1*‐dependent translational suppression and hyposynchrony of neural activity via phosphorylation of eIF2α and eEF2

**DOI:** 10.1002/jcp.30754

**Published:** 2022-04-17

**Authors:** Simon Lizarazo, Yeeun Yook, Nien‐Pei Tsai

**Affiliations:** ^1^ Department of Molecular and Integrative Physiology, School of Molecular and Cellular Biology University of Illinois at Urbana‐Champaign Urbana Illinois USA; ^2^ Neuroscience Program University of Illinois at Urbana‐Champaign Urbana Illinois USA

**Keywords:** eEF2, eIF2α, *Fmr1*, PP1, PP2A, translation

## Abstract

Alzheimer's disease (AD) is the most common cause of dementia, with the accumulation of amyloid beta peptide (Aβ) being one of the main causes of the disease. Fragile X mental retardation protein (FMRP), encoded by fragile X mental retardation 1 (*Fmr1*), is an RNA‐binding protein that represses translation of its bound mRNAs or exerts other indirect mechanisms that result in translational suppression. Because the accumulation of Aβ has been shown to cause translational suppression resulting from the elevated cellular stress response, in this study we asked whether and how *Fmr1* is involved in Aβ‐induced translational regulation. Our data first showed that the application of synthetic Aβ peptide induces the expression of *Fmr1* in cultured primary neurons. We followed by showing that *Fmr1* is required for Aβ‐induced translational suppression, hyposynchrony of neuronal firing activity, and loss of excitatory synapses. Mechanistically, we revealed that *Fmr1* functions to repress the expression of phosphatases including protein phosphatase 2A (PP2A) and protein phosphatase 1 (PP1), leading to elevated phosphorylation of eukaryotic initiation factor 2‐α (eIF2α) and eukaryotic elongation factor 2 (eEF2), and subsequent translational suppression. Finally, our data suggest that such translational suppression is critical to Aβ‐induced hyposynchrony of firing activity, but not the loss of synapses. Altogether, our study uncovers a novel mechanism by which Aβ triggers translational suppression and we reveal the participation of *Fmr1* in altered neural plasticity associated with Aβ pathology. Our study may also provide information for a better understanding of Aβ‐induced cellular stress responses in AD.

## Graphical abstract

The accumulation of Aβ causes translational suppression resulting from elevated cellular stress response but the mechanism is unclear. We showed that fragile X mental retardation protein (FMRP), an RNA‐binding protein commonly involved in autism spectrum disorders, can be induced by Aβ and contribute to Aβ‐dependent translational suppression and network hyposynchronicity. Our study may provide information for a better understanding of Aβ‐induced cellular stress responses in Alzheimer's disease.

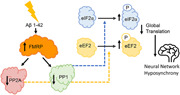

## 1 INTRODUCTION

Many cellular phenotypes observed in Alzheimer's disease (AD), including those with an accumulation of amyloid beta (Aβ), are associated with an elevation of cellular stress and subsequent stress responses (Briggs et al., 2017; Endres & Reinhardt, 2013). Those stress responses comprise a set of evolutionarily conserved mechanisms that help the cell adapt to disturbances. However, when attempts to cope with the disturbances fail or when the disturbances last for an extended period of time, the stress response can trigger cell degeneration and eventually cell death. Understanding cellular stress and the stress response in AD has the potential to facilitate the development of new therapies to ameliorate neurodegeneration. One common cellular stress response occurs through translational suppression (Appenzeller‐Herzog & Hall, 2012; Paschen et al., 2007). Pharmacologically reducing cellular stress‐associated translational suppression appears to be beneficial in AD; it improves neuronal function in an AD mouse model (Ma et al., 2013; Yang et al., 2016). However, our knowledge of the molecular regulation underlying translational suppression in AD is limited. To approach this question, we study *fragile X mental retardation 1* (*Fmr1*).

*Fmr1* is absent in the most common cause of intellectual disability and autism, fragile X syndrome (FXS). *Fmr1* encodes fragile X mental retardation protein (FMRP), an RNA‐binding protein that often represses the translation of its bound mRNAs (Ashley et al., 1993). In addition to binding to mRNAs, FMRP also exerts other indirect mechanisms that result in translational suppression (Lai et al., 2020; Valdez‐Sinon et al., 2020). Previous studies have indicated that the mRNA‐encoding amyloid precursor protein (APP) is one of the direct binding targets of FMRP; this led to the discovery that impaired APP expression might contribute to the deficits seen in FXS (Westmark et al., 2016). However, the knowledge of whether *Fmr1* inversely contributes to APP‐, or Aβ‐associated neurodegeneration remains elusive.

Extensive studies have demonstrated the role of *Fmr1* in regulating neural network activity and synapse numbers (Jewett et al., 2018; Liu et al., 2017, 2021; Tsai et al., 2017), which are known to be impaired in AD (Sheng et al., 2016). However, understanding whether and how *Fmr1* participates in Aβ pathology has been complicated (Hamilton et al., 2014; Renoux et al., 2014), potentially due to compensatory or feedback mechanisms resulting from chronic elevation of Aβ that occludes precise evaluation. To address this question, we employed an acute model of Aβ pathology using synthetic Aβ peptides in cultured primary cortical neurons. Using this system, we observed an elevation of both *Fmr1* and *Fmr1*‐dependent translational suppression in cultures treated with Aβ. We further showed that this translational suppression is induced by phosphorylation of eukaryotic translation initiation factor 2‐α (eIF2α) and eukaryotic translation elongation factor 2 (eEF2), mediated by an *Fmr1*‐dependent reduction of protein phosphatase 1 (PP1) and protein phosphatase 2A (PP2A), respectively, upon treatment with Aβ. Physiologically, we showed that such translational suppression is crucial to the hyposynchrony of neuronal firing activity, but not the loss of synapses. In summary, our study reveals a novel mechanism by which Aβ induces translational suppression to modulate neural activity. Our study also introduces *Fmr1* as a potential key molecule that contributes to cellular stress‐associated translational suppression in Aβ pathology. Building on existing tools and substantial knowledge of *Fmr1*, our research may open new avenues for the study of AD‐associated cognitive decline and memory impairment from the effects of *Fmr1*.

## 2 MATERIALS AND METHODS

The WT, *Fmr1* KO, and APP/PS1 mice in C57BL/6J background were obtained from The Jackson Laboratory. Trio breeding was conducted throughout the study. Both male and female mice were used to prepare mixed‐sex cultures. All animal procedures were performed in accordance with our institutional animal care committee's regulations. All experimental protocols involving mice were performed in accordance with the guidelines and regulations set forth by the Institutional Animal Care and Use Committee at the University of Illinois at Urbana‐Champaign.

### 2.1 Reagents

Amyloid beta (Aβ) 1−42 and scrambled Aβ peptide were from rPeptide. Dimethyl sulfoxide (DMSO) was from Fisher Scientific. Okadaic acid was from Sigma. The antibodies used in this study were purchased from Santa Cruz Biotechnology (anti‐PSD95), GenScript Corporation (anti‐Gapdh), Millipore (anti‐puromycin), Abcam (anti‐synapsin‐I, anti‐PSD95, anti‐p‐eIF4E, and anti‐MAP2) and Cell Signaling (anti‐eIF2A, anti‐p‐eIF2A, anti‐eIF4E, anti‐PDI, anti‐FMRP, anti‐eEF2, anti‐p‐eEF2, anti‐PP1, anti‐PP2A‐A, anti‐PP2A‐B, and anti‐PP2A‐C). Horseradish Peroxidase (HRP)‐conjugated secondary antibodies were from Cell Signaling and Jackson ImmunoResearch.

### 2.2 Real‐time quantitative reverse transcription PCR (RT‐qPCR)

After treatments, the total RNA from cultured neurons was obtained with TRIzol reagent (Life Technologies). Reverse transcription was performed with Photoscript reverse transcriptase (New England Biolab) and the real‐time PCR was performed with Thermo Scientific Maxima SYBR Green reagent. The primers used in this study were: *Fmr1*, 5’‐GAG ATC GTG GAC AAG TCA GGA G‐3' and 5'‐CTT CAG AGG AGT TAG GTC CAA CC‐3'; *Actin*, 5'‐CCT GTG CTG CTC ACC GAG GC‐3' and 5'‐GAC CCC GTC TCT CCG GAG TCC ATC‐3'.

### 2.3 Western blot analysis

Protein samples were separated by gel electrophoresis as described previously (Eagleman et al., 2021). After this, the gel was transferred onto a polyvinylidene fluoride membrane. The membrane was blocked with 1% bovine serum albumin solution in Tris‐buffered saline Tween‐20 buffer (TBST; 20 mM Tris pH 7.5, 150 mM NaCl, 0.1% Tween 20) and further incubated overnight with the corresponding primary antibody at 4°C. The following day, after three washes for 10 min each with TBST at room temperature, the membrane was incubated with the respective HRP‐conjugated secondary antibody in 5% nonfat milk in TBST for 1 h, followed by three more washes for 10 min each with TBST. Finally, the membrane was developed with an enhanced chemiluminescence reagent and detected by iBright imaging system (ThermoFisher). To quantify the puromycin signal, we measured the entire area of the smear (which spanned from slightly above 40 to 250 kDa), followed by subtracting the background (an area on the membrane without proteins). We then normalized this value relative to the GAPDH signal obtained from the same membrane, before comparing the fold change relative to the control treatment.

### 2.4 Multielectrode array (MEA) recording

All the MEA recordings were done using an Axion Muse 64‐channel system in single well MEAs (M64‐GL1‐30Pt200, Axion Biosystems) inside a 5% CO_2_, 37°C incubator. Field potentials (voltage) at each electrode relative to the ground electrode were recorded with a sampling rate of 25 kHz. After 30 min of recording the baseline (before), drug(s) indicated in each experiment was added, and the MEA dish was immediately put back into the incubator. Following the treatments, another 30 min of recording was perform (after). Due to changes in network activity caused by physical movement of the MEA, only the last 15 min of each recording were used in data analyses as performed previously (Jewett et al., 2016). AxIS software (Axion Biosystems) was used for the extraction of spontaneous spikes from the raw electrical signal obtained from the Axion Muse system. After filtering, a threshold of ±6 standard deviations was independently set for each channel; activity exceeding this threshold was counted as a spike. The synchronicity of spontaneous spikes is accessed by the synchrony index, which was computed through AxIS software, based on a previously published algorithm (Eggermont, 2006), by taking the cross‐correlation between two spike trains, removing the portions of the cross‐correlogram that are contributed by the auto‐correlations of each spike train, and reducing the distribution to a single metric. A value of 0 corresponds to no synchrony and a value of 1 corresponds to perfect synchrony.

### 2.5 Immunocytochemistry

Immunocytochemistry was done as previously described (Lee et al., 2021). In brief, primary neurons were cultured on poly‐d‐lysine‐coated coverslips. At DIV 12−14 after treatment, cells were fixed with ice‐cold buffer (4% paraformaldehyde and 5% sucrose in PBS). Then cells were permeabilized with an additional incubation with 0.5% Triton X‐100 in PBS. After permeabilization, incubation with anti‐PSD95, anti‐synapsin‐I, and anti‐MAP2 antibodies was performed overnight at 4°C. The following day, after three washings with PBS, fluorescen‐conjugated secondary antibodies were added, and the cells were incubated protected from light during 2 h. The cells were further washed three times more and the coverslips were mounted using a mounting medium (Fisher Scientific). The coverslips were observed under Zeiss LSM 700 Confocal Microscope with ×40 magnification. Pinhole was set to 1 airy unit for all experiments and settings were kept with the same laser and scanning configurations. Synapse number was quantified by measuring the colocalization of presynaptic and postsynaptic markers in secondary dendrites. ImageJ software with SynapCountJ plugin was used for data analysis.

### 2.6 Statistical analysis

The variability is commonly observed across different batches of primary cultures. To accommodate the variability, the cultures made from the same litter of mice through one dissection were used for all treatment groups in each experiment. G* power was used to perform power analysis. Expected effect sizes were based on our previously published studies (Eagleman et al., 2021; Lodes et al., 2021). Sample sizes for western blot, MEA recording, and immunocytochemistry were equal or greater than the suggested size of 4, 6, and 11, respectively, for a power of 0.8 and a Type I error (alpha) of 0.05. Outliers were determined using Grubbs' test. For multiple comparisons, two‐way ANOVA with post hoc Tukey HSD (Honestly Significant Difference) test were performed. For experiments where only two conditions were performed, the Student's *t* test was used. In all figures, error bars represent SEM and ^∗^ *p* < 0.05, ^∗∗^ *p* < 0.01.

## 3 RESULTS

### 3.1 Amyloid beta induces *Fmr1* and *Fmr1*‐dependent translational suppression

**Figure 1:**
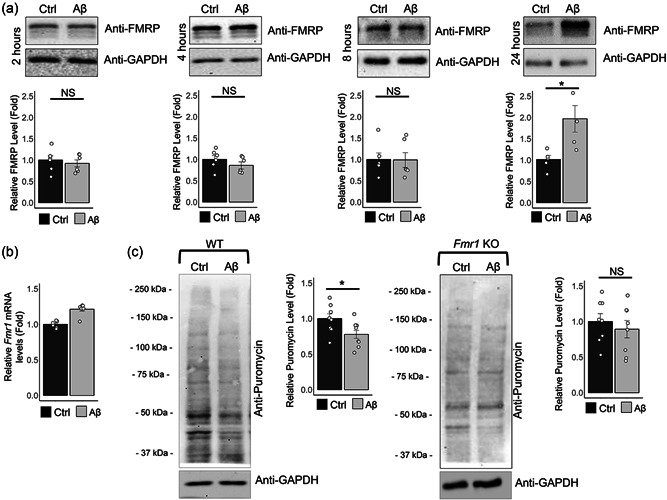
Amyloid beta induces*Fmr1* and*Fmr1*‐dependent translational suppression. (a) Representative western blots and quantifications of FMRP and GAPDH from WT cortical neuron cultures treated with amyloid beta 1−42 (Aβ; 1 µM) or scrambled Aβ peptide (Ctrl, 1 µM) for 2, 4, 8, or 24 h at DIV 12−14. (*n* = 5−6 from three independent cultures). (b) Quantitative real‐time RT‐PCR of *Fmr1* mRNA normalized to *Actin* mRNA from WT cortical neuron cultures treated with amyloid beta 1−42 (Aβ; 1 µM) or scrambled Aβ peptide (Ctrl, 1 µM) for 24 h at DIV 12−14. (*n* = 6 from three independent cultures). (c) Representative western blots and quantifications of puromycin and GAPDH from WT and *Fmr1* KO cortical neuron cultures treated with amyloid beta 1−42 (Aβ; 1 µM) or scrambled Aβ peptide (Ctrl, 1 µM) for 24 h at DIV 12−14 (*n* = 8 from three independent cultures). No data points were removed after the Grubbs’ outlier test. Student's *t* test was used. Data are represented as mean ± SEM with *p < 0.05, ns, nonsignificant.

To determine the functional interaction between Aβ and *Fmr1*, we first examined whether Aβ could mediate the expression of FMRP. To do so, we utilized synthetic Aβ peptide 1−42 (Aβ42), one of the neurotoxic forms of Aβ (Müller et al., 2017). Primary cortical neurons from wild‐type (WT) mice at days‐in‐vitro (DIV) 12–14 were treated with either Aβ42 or a scrambled Aβ control peptide (1 µM) for the duration of 2, 4, 8, or 24 h. As shown in Figure 1a, the levels of FMRP did not change following the treatments of Aβ42 for 2, 4, or 8 h but increased significantly after the treatment for 24 h. This elevation can also be seen in a well‐established AD mouse model, the APP/PS1 mice, at 12 weeks of age (Figure S1), which is around the time when early cellular phenotypes start to appear in these mice (Cheng et al., 2020; Zhurakovskaya et al., 2019). The relatively slow response in cultures suggests a possibility that the elevation of FMRP might occur at the mRNA level. To test this possibility, we employed RT‐qPCR to assess the levels of *Fmr1*. As shown in Figure 1b, the levels of *Fmr1* are significantly elevated following the treatment with Aβ42 for 24 h. These data suggest that Aβ42 promotes the expression of *Fmr1* both in APP/PS1 mice and in cultured cortical neurons.

FMRP is an RNA‐binding protein involved in the stability, maturation, transport, and translation of its bound mRNA (D'Annessa et al., 2019). It also contributes to global mRNA translation through other indirect mechanisms (Lai et al., 2020; Valdez‐Sinon et al., 2020). Because Aβ42 is known to trigger translational suppression and such translational suppression has been indicated as contributing to neurodegeneration (Briggs et al., 2017; de la Monte, 2012; Endres & Reinhardt, 2013; Mukherjee & Soto, 2011), we asked whether *Fmr1* contributes to Aβ42‐induced translational suppression. To this end, we treated both WT and *Fmr1* KO (knockout) primary cortical neuron cultures with Aβ42 for 24 h, labeled newly synthesized proteins with puromycin (10 µg/ml) during the last 30 min, and followed this with western blot analysis with an antipuromycin antibody. As shown in Figure 1c, incubation with Aβ42 significantly reduces translation in WT but not in *Fmr1* KO neurons. Taken together, our data suggest that *Fmr1* is required for Aβ42‐induced translational suppression.

### 3.2 Amyloid beta induces hyposynchrony of firing activity and reduces the number of synapses in an *Fmr1*‐dependent manner

**Figure 2:**
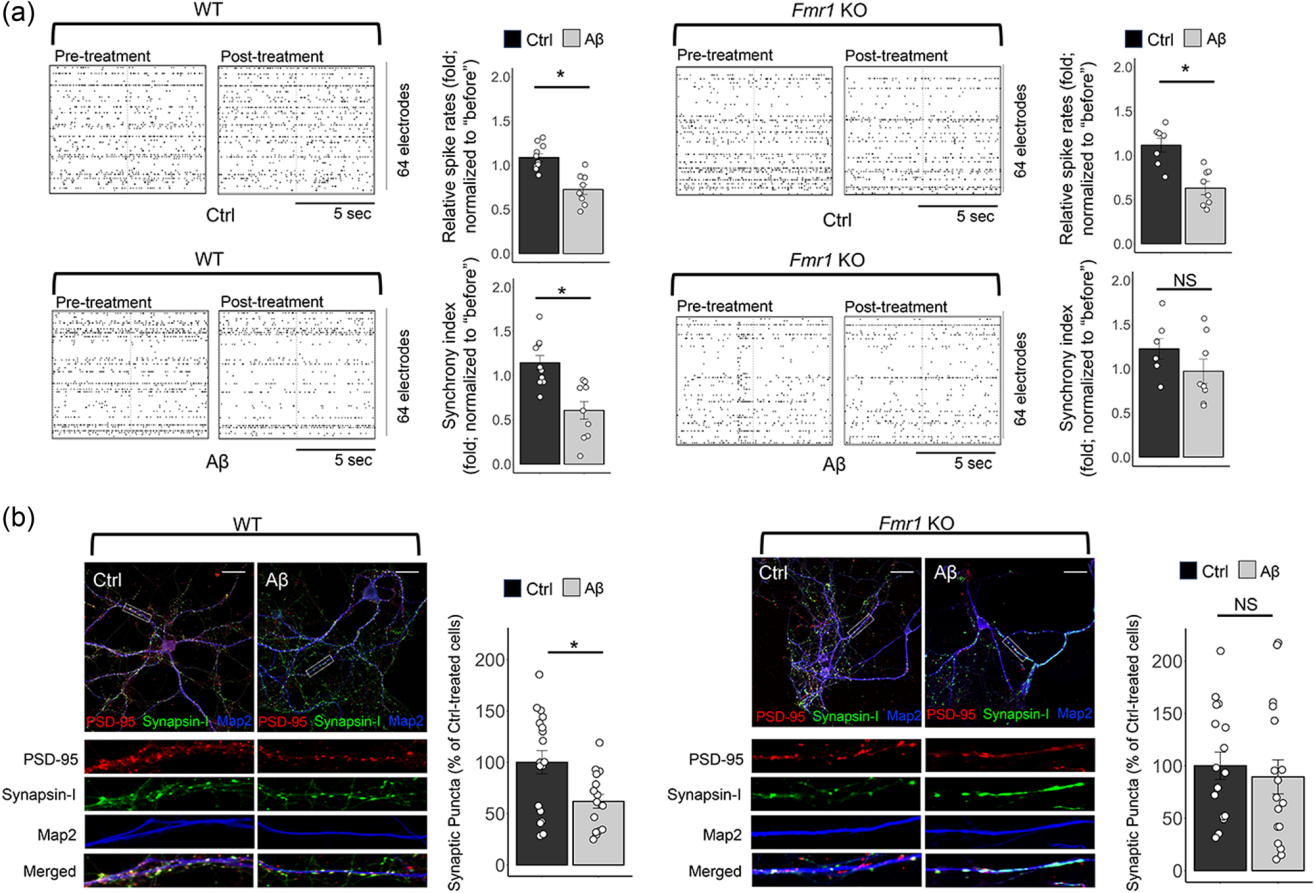
Amyloid beta induces*Fmr1*‐dependent hyposynchrony of neural network activity and reduction of synapse number. (a) Representative raster plots of spontaneous spikes from WT and *Fmr1* cortical neuron cultures treated with amyloid beta 1−42 (Aβ; 1 µM) or scrambled Aβ peptide (Ctrl, 1 µM) for 24 h at DIV 12−14. Quantification of spontaneous spike rate and synchrony index by comparing “after treatment” to “before treatment,” from the same culture was shown on the right. (*n* = 6−8 independent cultures after removing one culture from spike rate and synchrony index analyses in WT cultures treated with amyloid beta, and one culture from spike rate and synchrony index analyses in *Fmr1* cultures treated with scrambled peptide following the Grubbs' outlier test.) (b) Immunocytochemistry showing postsynaptic marker PSD‐95 (red), presynaptic marker synapsin‐I (green), dendritic marker Map2 (blue), and colocalization of PSD‐95 and synapsin‐I from dissociated WT and *Fmr1* KO cortical neuron cultures treated with amyloid beta 1−42 (Aβ; 1 µM) or scrambled Aβ peptide (Ctrl, 1 µM) for 24 h at DIV 12−14. Representative secondary dendrites are displayed and quantification of colocalized synapses as relative percentage of number of synapses was shown. (For WT, data were collected from two independent cultures with *n* = 10 and 7 cells treated with amyloid beta and *n* = 11 and 7 cells treated with scrambled peptide. For *Fmr1* KO, data were collected from two independent cultures with *n* = 8 and 9 cells treated with amyloid beta and *n* = 8 and 9 cells treated with scrambled peptide. One cell in WT cultures treated with amyloid beta was removed following the Grubbs' outlier test.) Student's *t* test was used. Scale bar: 10 µm. Data are represented as mean ± SEM with *p < 0.05, ns, nonsignificant.

To explore whether *Fmr1* is involved in other Aβ42‐induced alterations of neuronal functions, we employed an ME) recording system to assess extracellular spontaneous spikes from WT or *Fmr1* KO cultures following treatment with Aβ42 for 24 h. As shown in Figure 2a, Aβ42 induced a significant reduction in the rate of spontaneous spikes in both WT and *Fmr1* KO cultures. However, when we evaluated the firing pattern of spontaneous spikes, we found that Aβ42 induced a significant reduction in the synchronicity of spikes in WT but not in *Fmr1* KO cultures. Reduced synchronicity of neuronal firing activity has been previously demonstrated in a model of AD (Ranasinghe et al., 2021), and our data suggest that *Fmr1* is required for the process.

Hyposynchrony of spikes can be in part attributed to a reduced number of excitatory synapses (Golomb & Hansel, 2000), which is an early hallmark of neurodegeneration in AD (Sheng et al., 2016). Because multiple studies have demonstrated the role of *Fmr1* in suppressing synapse numbers, in part through translational suppression (Comery et al., 1997; Huebschman et al., 2020; Pfeiffer et al., 2010; Tsai et al., 2017; Zang et al., 2013), we asked whether *Fmr1* participates in Aβ42‐induced loss of synapses. To answer this question, we performed immunocytochemistry to quantify synapse numbers in WT and *Fmr1* KO cortical neuron cultures treated with Aβ42 or a control peptide for 24 h. Following the treatments, the neurons were fixed and stained for the presynaptic marker synapsin‐I and the postsynaptic marker postsynaptic density protein 95 (PSD‐95) to measure colocalization of pre‐ and postsynaptic puncta, as we have done recently (Lee et al., 2021). As shown, while the basal levels of PSD‐95 and Synapsin‐I were not significantly different between WT and *Fmr1* KO neurons (Figure S2), in comparison to the control peptide, Aβ42 induced a reduction in synapse number in WT cultures but this reduction was not observed in *Fmr1* KO cultures (Figure 2b). Altogether, our data suggest that *Fmr1* is required for Aβ42‐induced hyposynchrony of neural network activity and the reduction of synapse numbers.

### 3.3 *Fmr1* mediates amyloid beta‐induced phosphorylation of eIF2α

**Figure 3:**
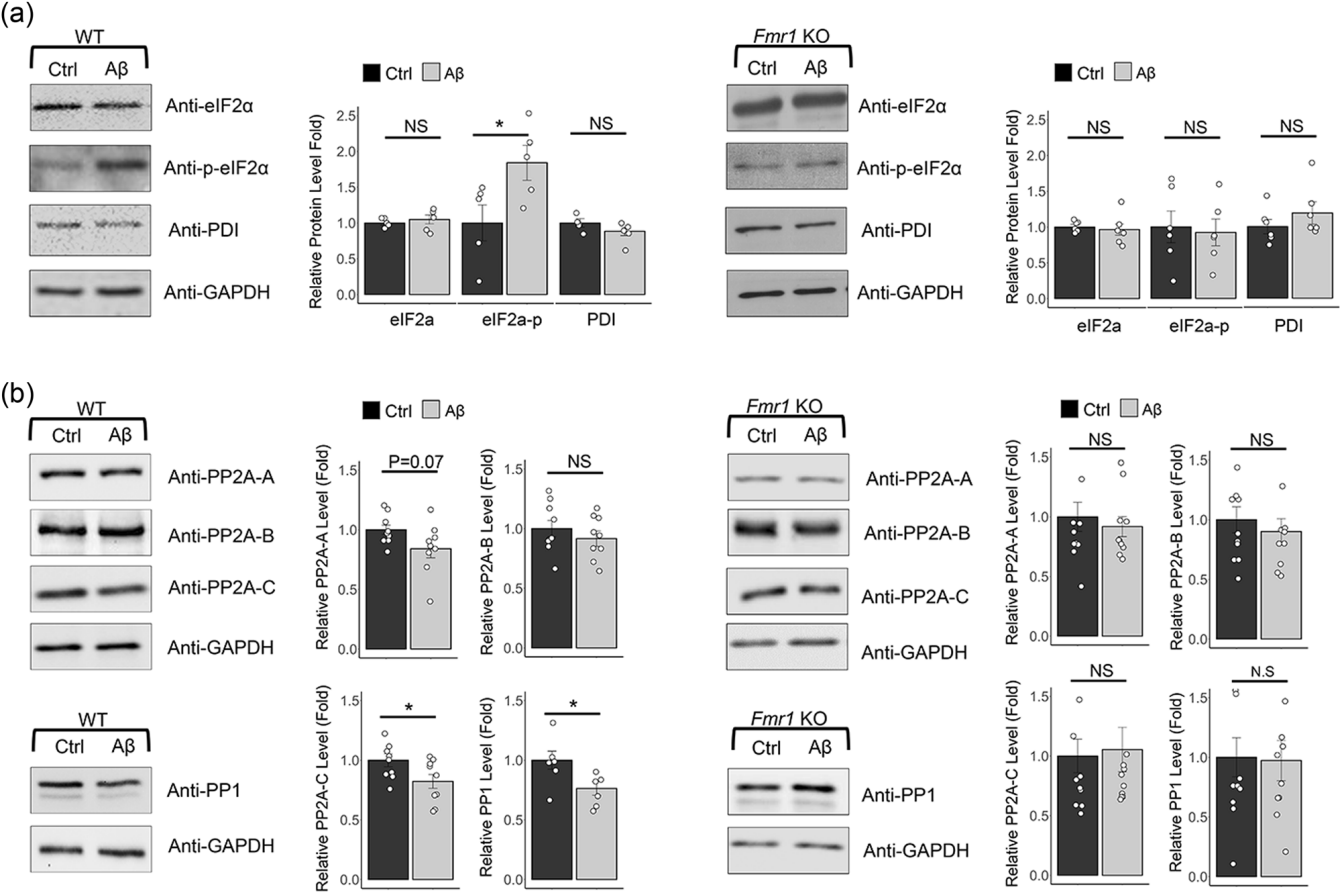
Amyloid beta induces phosphorylation of eIF2α in an*Fmr1*‐dependent manner. (a) Representative western blots and quantifications of eIF2α, p‐eIF2α, PDI, and GAPDH from WT and *Fmr1* KO cortical neuron cultures treated with amyloid beta 1−42 (Aβ; 1 µM) or scrambled Aβ peptide (Ctrl, 1 µM) for 24 h at DIV 12−14. (*n* = 5−6 independent cultures. No data points were removed after the Grubbs' outlier test.) (b) Representative western blots and quantifications of PP2A‐A, PP2A‐B, PP2A‐C, PP1, and GAPDH from WT and *Fmr1* KO cortical neuron cultures treated with amyloid beta 1−42 (Aβ; 1 µM) or scrambled Aβ peptide (Ctrl, 1 µM) for 24 h at DIV 12−14. (*n* = 6−9 from three independent cultures. One *Fmr1* KO culture treated with amyloid beta was removed from analyses for PP2A‐A, PP2A‐B, and PP1 following the Grubbs' outlier test.) Student's *t* test was used. Data are represented as mean ± SEM with *p < 0.05; ns, nonsignificant.

In AD, the accumulation of Aβ has been shown to cause exaggerated translational suppression (Beckelman et al., 2019; Ding et al., 2005; Hernández‐Ortega et al., 2016; Langstrom et al., 1989; Oliveira et al., 2021; Radford et al., 2015; Sajdel‐Sulkowska & Marotta, 1984), in part through phosphorylation of eIF2α (Ma et al., 2013). We therefore asked whether *Fmr1* is involved in the phosphorylation of eIF2α following treatment with Aβ42. As shown in Figure 3a, we observed an increase in eIF2α phosphorylation in WT neurons but not in *Fmr1* KO neurons. No changes were observed in phosphorylation of eukaryotic translation initiation factor 4E (eIF4E) (Figure S3), another altered signaling pathway related to translational control in AD (Ghosh et al., 2020). There are also no changes in the levels of protein disulfide isomerase (PDI), a common endoplasmic reticulum (ER) stress marker in both genotypes, suggesting a specific role for *Fmr1* in regulating eIF2α phosphorylation rather than altered cellular stress responses in *Fmr1* KO neurons.

Because FMRP functions as a translational suppressor for selective mRNAs, we suspect that FMRP represses the expression of certain phosphatases for eIF2α to elevate eIF2α phosphorylation. To this end, we evaluated the expression of serine/threonine phosphatases, such as PP1 and PP2A, which are known targets of FMRP (Darnell et al., 2011). As shown in Figure 3b, we found that treatment with Aβ42 significantly downregulates the levels of the catalytic subunit of PP2A (PP2A‐C) and PP1 in WT but not in *Fmr1* KO neurons. These data indicate the possibility that impaired downregulation of PP2A‐C and PP1 is responsible for impaired eIF2α phosphorylation and translational suppression in *Fmr1* KO neurons.

### 3.4 PP2A and PP1 differentially regulate Aβ42‐induced eIF2α phosphorylation and translational suppression

**Figure 4:**
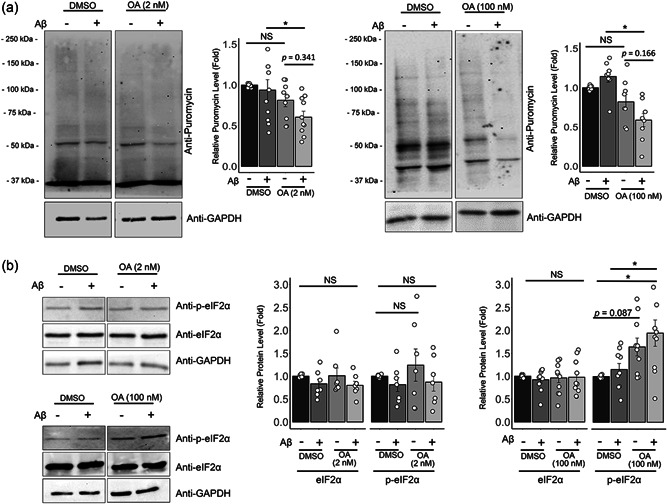
PP2A and PP1 differentially regulate Aβ42‐induced eIF2α phosphorylation and translational suppression. (a) Representative western blots and quantifications of puromycin and GAPDH from *Fmr1* KO cortical neuron cultures treated with amyloid beta 1−42 (Aβ; 1 µM) or scrambled Aβ peptide (Ctrl, 1 µM) for 24 h and with vehicle (DMSO) or okadaic acid (2 nM or 100 nM) for the last hour at DIV 12−14. (*n* = 7−9 independent cultures after removing one culture treated with scrambled peptide + 2 nM okadaic acid following the Grubbs' outlier test.) Two‐way ANOVA with Tukey test were used. (For the left panel, interaction: *F*_1,35_ = 0.724, *p* = 0.400; drug effect: *F*_1,35_ = 9.624, *p* = 0.004; peptide effect: *F*_1,35_ = 2.426, *p* = 0.128. For the right panel, interaction: *F*_1,28_ = 5.969, *p* = 0.021; drug effect: *F*_1,28_ = 22.689, *p* = 0.00005; peptide effect: *F*_1,28_ = 0.335, *p* = 0.567.) (b) Representative western blots and quantifications of eIF2α, p‐eIF2α, and GAPDH from *Fmr1* KO cortical neuron cultures treated with amyloid beta 1−42 (Aβ; 1 µM) or scrambled Aβ peptide (Ctrl, 1 µM) for 24 h and with vehicle (DMSO) or okadaic acid (2 or 100 nM) for the last hour at DIV 12−14. (*n* = 7−10 independent cultures after removing one culture treated with amyloid beta + 100 nM okadaic acid for the analyses of eIF2α and p‐eIF2α following the Grubbs' outlier test.) Two‐way ANOVA with Tukey test were used. (For OA at 2 nM, the left panel, interaction: *F*_1,24_ = 0.041, *p* = 0.842; drug effect: *F*_1,24_ = 0.010, *p* = 0.920; peptide effect: *F*_1,24_ = 3.093, *p* = 0.091. For OA at 2 nM, the right panel, interaction: *F*_1,24_ = 0.189, *p* = 0.668; drug effect: *F*_1,24_ = 0.471, *p* = 0.499; peptide effect: *F*_1,24_ = 1.673, *p* = 0.208. For OA at 100 nM, the left panel, interaction: *F*_1,35_ = 0.274, *p* = 0.604; drug effect: *F*_1,35_ = 0.005, *p* = 0.941; peptide effect: *F*_1,35_ = 0.122, *p* = 0.729. For OA at 100 nM, the right panel, interaction: *F*_1,35_ = 0.152, *p* = 0.698; drug effect: *F*_1,35_ = 14.641, *p* = 0.0005; peptide effect: *F*_1,35_ = 1.467, *p* = 0.234.) Data are represented as mean ± SEM with *p < 0.05, ns, nonsignificant.

To validate our hypothesis that the downregulation of PP2A‐C and PP1 is crucial to Aβ42‐induced, *Fmr1*‐dependent translational suppression, we proposed to pharmacologically inhibit PP2A and PP1 in *Fmr1* KO neurons with the intention of restoring translational suppression. To this end, we treated *Fmr1* KO cortical neuron cultures with Aβ42 for 24 h, and with okadaic acid (OA) at either 2 nM to inhibit PP2A or at 100 nM to inhibit both PP2A and PP1 (Holmes et al., 1990) during the last hour. To label newly synthesized proteins, puromycin was added during the last 30 min of the Aβ42 treatment as illustrated in Figure 1c. As shown in Figure 4a, while OA at both 2 and 100 nM showed some basal effects toward reduction of protein synthesis, it was able to restore Aβ42‐induced translational suppression in *Fmr1* KO neurons. Although OA at 2 nM slightly reduced basal protein synthesis, it did not significantly exert further effects toward protein synthesis following Aβ42 treatment in WT neurons (Figure S4), supporting our observation that PP2A primarily functions downstream of Aβ42 on translational suppression. These results described above suggest the participation of PP2A in Aβ42‐induced translational suppression, although we were unable to confirm the extent to which PP1 is involved because OA at 100 nM inhibits both PP2A and PP1. However, when we evaluated the phosphorylation of eIF2α, we surprisingly found that OA at 100 nM, but not at 2 nM, was able to elevate the levels of eIF2α phosphorylation following Aβ42 treatment in *Fmr1* KO neurons (Figure 4b). OA at 100 nM even exerted a strong effect on eIF2α phosphorylation in the absence of Aβ42 treatment, suggesting an Aβ42‐independent effect. Taken together, these data suggest that PP1 is involved in Aβ42‐induced translational suppression potentially via eIF2α phosphorylation. On the other hand, PP2A is likely mediating this translational suppression through an eIF2α phosphorylation‐independent mechanism, and we aimed to characterize that next.

### 3.5 *Fmr1* mediates amyloid beta‐induced phosphorylation of eEF2 through PP2A

**Figure 5:**
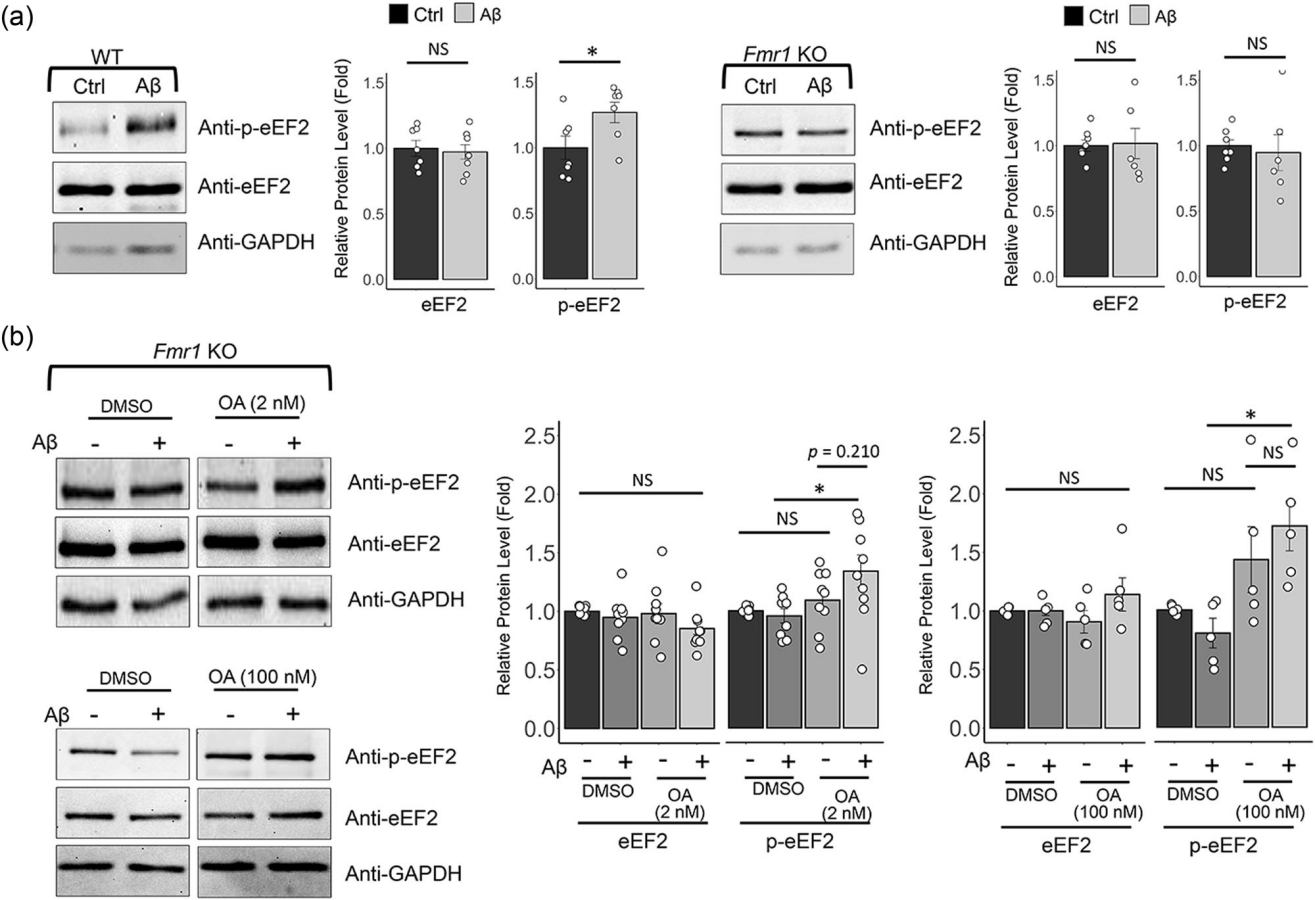
*Fmr1* mediates amyloid beta‐induced phosphorylation of eEF2 through PP2A. (a) Representative western blots and quantifications of p‐eEF2, eEF2, and GAPDH from WT and *Fmr1* KO cortical neuron cultures treated with amyloid beta 1−42 (Aβ; 1 µM) or scrambled Aβ peptide (Ctrl, 1 µM) for 24 h at DIV 12−14. (*n* = 6−7 independent cultures after removing one *Fmr1* KO culture treated with amyloid beta following the Grubbs' outlier test.) (b) Representative western blots and quantifications of eEF2, p‐eEF2, and GAPDH from *Fmr1* KO cortical neuron cultures treated with amyloid beta 1−42 (Aβ; 1 µM) or scrambled Aβ peptide (Ctrl, 1 µM) for 24 h and with vehicle (DMSO) or okadaic acid (2 or 100 nM) for the last hour at DIV 12−14. (*n* = 5−9 independent cultures after removing one culture treated with amyloid beta + DMSO for the analysis of p‐eEF2 following the Grubbs' outlier test.) Student's *t *test was used in (a) and two‐way ANOVA with Tukey test was used in (b). (For OA at 2 nM, the left panel, interaction: *F*_1,32_ = 0.438, *p* = 0.513; drug effect: *F*_1,32_ = 0.982, *p* = 0.329; peptide effect: *F*_1,32_ = 2.437, *p* = 0.128. For OA at 2 nM, the right panel, interaction: *F*_1,31_ = 2.689, *p* = 0.111; drug effect: *F*_1,31_ = 6.920, *p* = 0.013; peptide effect: *F*_1,31_ = 1.430, *p* = 0.241. For OA at 100 nM, the left panel, interaction: *F*_1,16_ = 1.727, *p* = 0.207; drug effect: *F*_1,16_ = 0.068, *p* = 0.797; peptide effect: *F*_1,16_ = 1.766, *p* = 0.202. For OA at 100 nM, the right panel, interaction: *F*_1,16_ = 1.671, *p* = 0.215; drug effect: *F*_1,16_ = 12.825, *p* = 0.003; peptide effect: *F*_1,16_ = 0.057, *p* = 0.814.) Data are represented as mean ± SEM with *p < 0.05, ns, nonsignificant.

In addition to eIF2α, the accumulation of Aβ has also been shown to cause translational suppression in part through phosphorylation of eEF2 (Beckelman et al., 2019). Because PP2A has been reported to participate in eEF2 dephosphorylation (Chang et al., 2018), we hypothesized that Aβ42 can induce eEF2 phosphorylation through *Fmr1* and PP2A. As shown in Figure 5a, treatment with Aβ42 elevated the levels of eEF2 phosphorylation in WT but not in *Fmr1* KO neurons, confirming the necessity of *Fmr1* in the process. Most importantly, OA at 2 nM was able to fully restore eEF2 phosphorylation in *Fmr1* KO neurons following the treatment with Aβ42 (Figure 5b). OA at 2 nM does not alter basal levels of eEF2 phosphorylation or exert significant effects toward eEF2 phosphorylation following Aβ treatment in WT neurons (Figure S5), supporting our claim that PP2A primarily functions downstream of Aβ42 on eEF2 phosphorylation. On the other hand, OA at 100 nM exerted a strong trend toward eEF2 phosphorylation even in the absence of Aβ42, suggesting a role of PP1 in basal eEF2 dephosphorylation independent of Aβ42. Altogether, our data suggest that, following treatment with Aβ42, elevated eEF2 phosphorylation is mediated primarily by PP2A, elevated eIF2α phosphorylation is likely mediated by PP1, and *Fmr1* functions to allow both pathways.

### 3.6 Inhibition of PP2A restores amyloid beta‐induced hyposynchrony of firing activity but not the reduction of synapse numbers in *Fmr1* KO neurons

**Figure 6:**
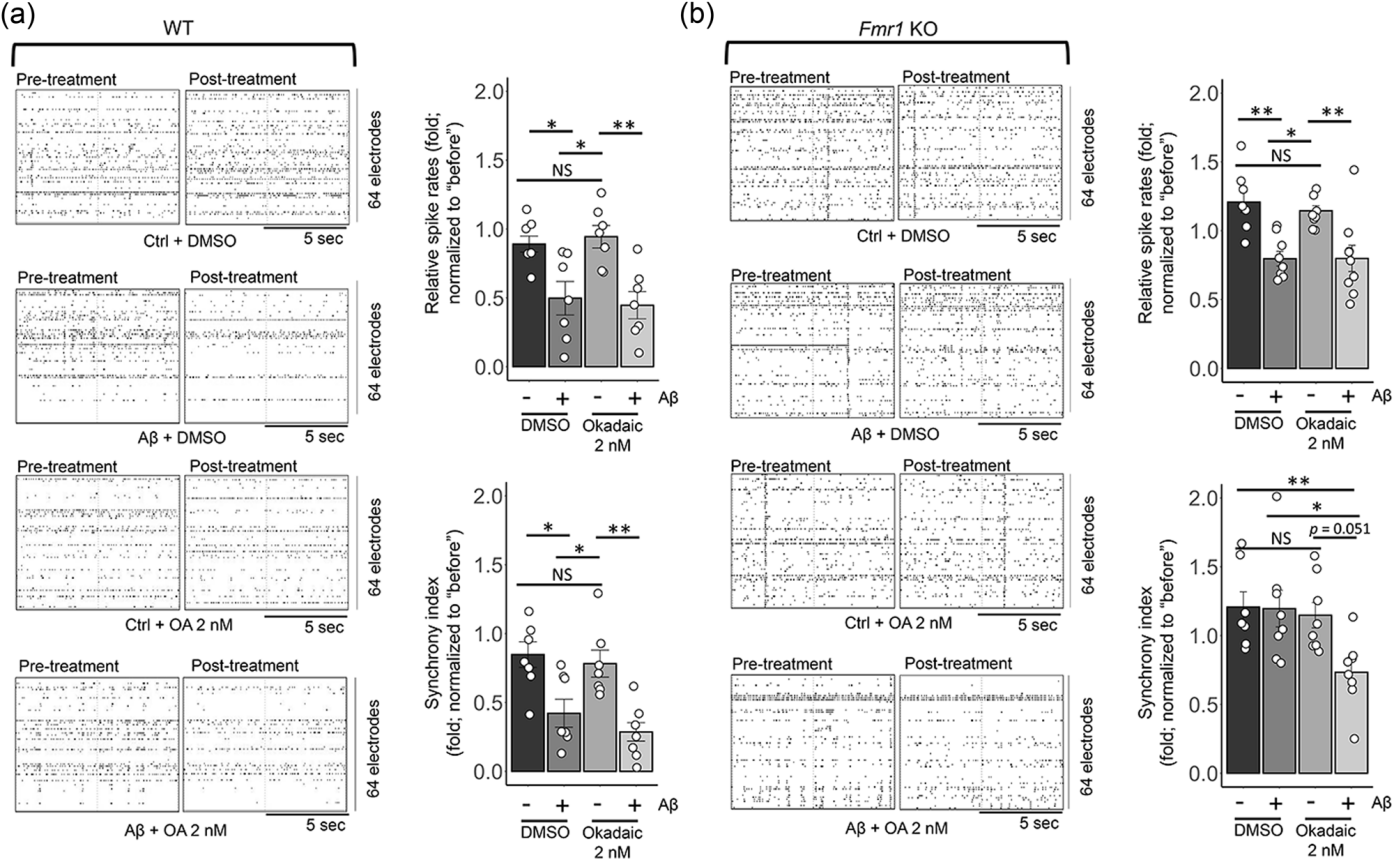
Inhibition of PP2A restores amyloid beta‐induced hyposynchrony of firing activity in *Fmr1*KO neurons. (a,b) Representative raster plots of spontaneous spikes from WT (a) and *Fmr1* KO (b) cortical neuron cultures treated with amyloid beta 1−42 (Aβ; 1 µM) or scrambled Aβ peptide (Ctrl, 1 µM) for 24 h and with vehicle (DMSO) or okadaic acid (2 nM) for the last hour at DIV 12−14. Quantification of spontaneous spike rate and synchrony index by comparing “after treatment” to “before treatment,” from the same culture was shown on the right. (*n* = 6−8 independent cultures after removing one *Fmr1* KO culture treated with scrambled peptide + DMSO for analyses of spike rate and synchrony index, one *Fmr1* KO culture treated with amyloid beta + DMSO for analyses of spike rate, and one *Fmr1* KO culture treated with scrambled peptide + okadaic acid for analyses of spike rate following the Grubbs' outlier test.) Two‐way ANOVA with Tukey test were used; for (a), top panel, interaction: *F*_1,24_ = 0.315, *p* = 0.580; drug effect: *F*_1,24_ = 0.000, *p* = 0.990; peptide effect: *F*_1,24_ = 22.904, *p* = 0.00007. For (a), bottom panel, interaction: *F*_1,24_ = 0.147, *p* = 0.704; drug effect: *F*_1,24_ = 1.222, *p* = 0.280; peptide effect: *F*_1,24_ = 25.825, *p* = 0.00003. For (b), top panel, interaction: *F*_1,28_ = 0.188, *p* = 0.668; drug effect: *F*_1,28_ = 0.131, *p* = 0.720; peptide effect: *F*_1,28_ = 25.948, *p* = 0.00002. For (a), bottom panel, interaction: *F*_1,27_ = 3.376, *p* = 0.077; drug effect: *F*_1,27_ = 5.660, *p* = 0.025; peptide effect: *F*_1,27_ = 4.037, *p* = 0.055. Data are represented as mean ± SEM with *p < 0.05; **p < 0.01, ns, nonsignificant.

**Figure 7:**
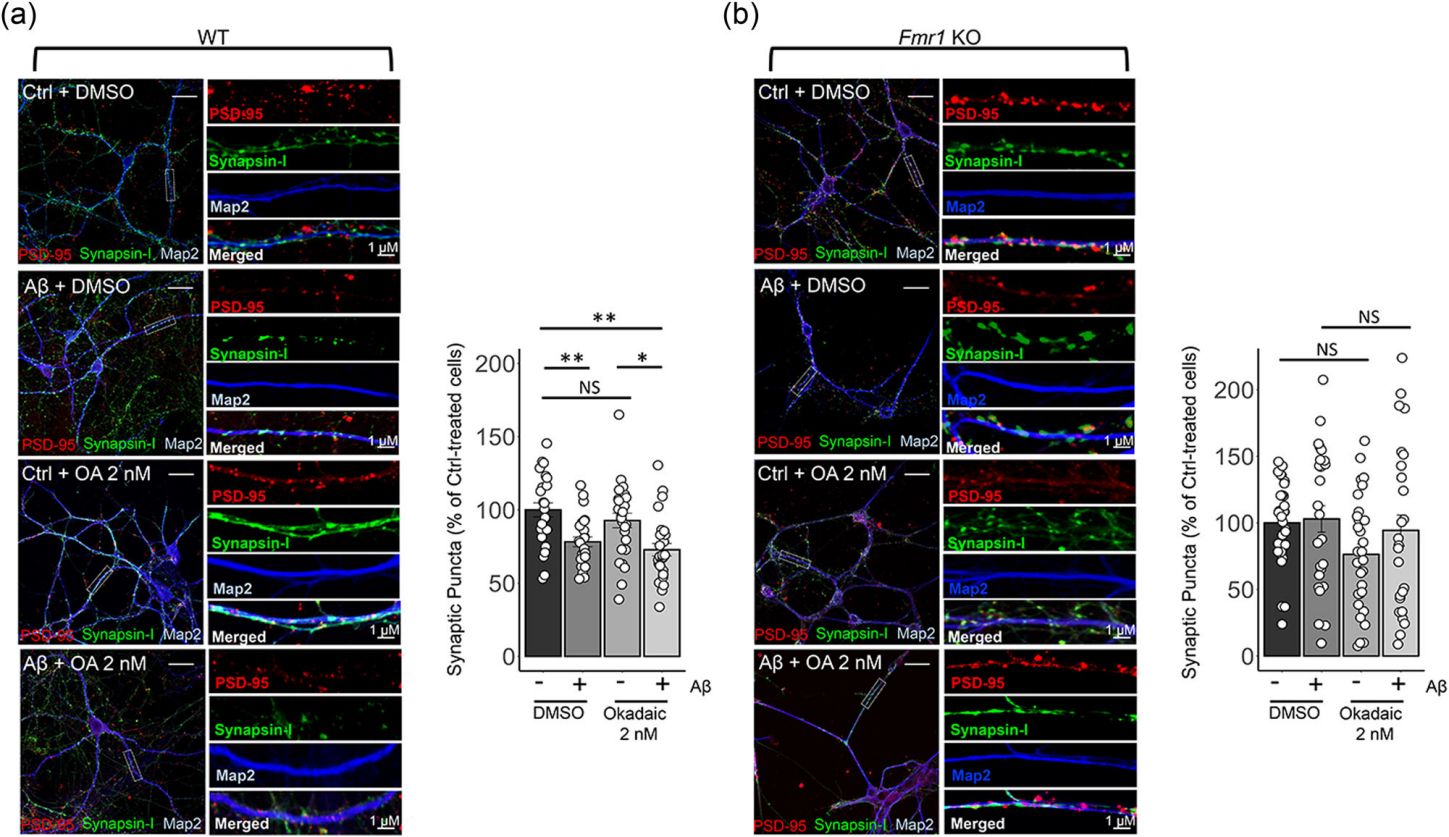
Inhibition of PP2A does not restore amyloid beta‐induced reduction of number of synapses in *Fmr1*KO neurons. (a,b) Immunocytochemistry showing postsynaptic marker PSD‐95 (red), presynaptic marker synapsin‐I (green), dendritic marker Map2 (blue), and colocalization of PSD‐95 and synapsin‐I from dissociated WT (a) or *Fmr1* KO (b) cortical neuron cultures treated with amyloid beta 1−42 (Aβ; 1 µM) or scrambled Aβ peptide (Ctrl, 1 µM) for 24 h and with vehicle (DMSO) or okadaic acid (2 nM) for the last hour at DIV 12−14. Representative secondary dendrites are displayed and quantification of colocalized synapses as relative percentage of number of synapses was shown. (For WT, data were collected from two independent cultures with *n* = 10 and 16 cells treated with scrambled peptide, *n* = 9 and 15 cells treated with s amyloid beta, *n* = 13 and 14 cells treated with scrambled peptide + okadaic acid, and *n* = 9 and 16 cells treated with s amyloid beta + okadaic acid. For *Fmr1* KO, data were collected from two independent cultures with *n* = 16 and 14 cells treated with scrambled peptide, *n* = 13 and 12 cells treated with s amyloid beta, *n* = 15 and 15 cells treated with scrambled peptide + okadaic acid, and *n* = 15 and 11 cells treated with s amyloid beta + okadaic acid. One cell in WT cultures treated with scrambled peptide + DMSO and one cell in WT cultures treated with amyloid beta + okadaic acid was removed following the Grubbs' outlier test.) Two‐way ANOVA with Tukey test were used; for (a), interaction: *F*_1,97_ = 0.040, *p* = 0.842; drug effect: *F*_1,97_ = 1.822, *p* = 0.180; peptide effect: *F*_1,97_ = 20.995, *p* = 0.00001. For (b), interaction: *F*_1,106_ = 3.306, *p* = 0.072; drug effect: *F*_1,106_ = 1.267, *p* = 0.263; peptide effect: *F*_1,106_ = 0.654, *p* = 0.421. Data are represented as mean ± SEM with *p < 0.05; **p < 0.01, ns, nonsignificant.

We suggested the role of *Fmr1* in allowing PP1‐dependent eIF2α phosphorylation (Figure 4) and PP2A‐dependent eEF2 phosphorylation (Figure 5) in Aβ42‐induced translational suppression. We then sought to determine whether PP2A or PP1 also participates in Aβ42‐induced, *Fmr1*‐dependent hyposynchrony of neural network activity and the reductions in synapse numbers that we observed (see Figure 2). However, because our data suggest that PP1 mediates basal dephosphorylation of eIF2α and eEF2 even in the absence of Aβ42 (Figures 4b, 5b) whereas PP2A appears to be more specific to Aβ42‐induced eEF2 dephosphorylation (Figure 5), we decided to focus on PP2A and asked whether inhibiting PP2A can restore the hyposynchrony of neural network activity and the reductions in synapse numbers in *Fmr1* KO neurons following treatment with Aβ42. As shown in Figures 6a, 7a, we showed that OA at 2 nM did not elicit an additional effect on already reduced synchronization neural network activity and synapse numbers in WT neurons following treatment with Aβ42. But importantly, we found that OA at 2 nM was able to fully restore the hyposynchrony of neural network activity without altering the rate of spontaneous spikes following treatments with Aβ42 in *Fmr1* KO neurons (Figure 6b). Interestingly, OA at 2 nM was unable to restore Aβ42‐induced reductions in synapse numbers in *Fmr1* KO neurons (Figure 7b), suggesting the likelihood that Aβ42‐induced, *Fmr1*‐dependent hyposynchrony of neural network activity and the reduction in synapse numbers are two independent events, and that PP2A is primarily involved in the former one.

## 4 DISCUSSION

**Figure 8:**
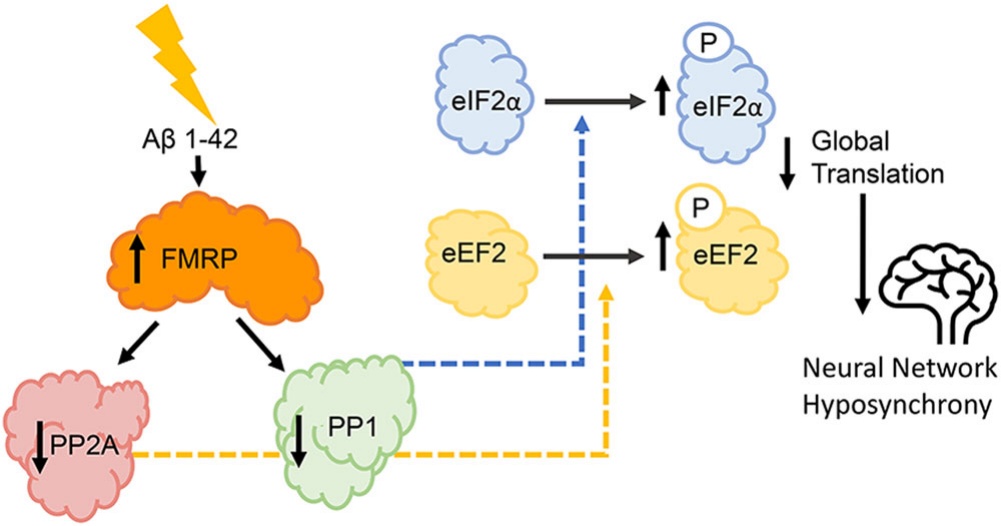
A working model describing the role of *Fmr1* in amyloid beta (Aβ) induced‐translational suppression.

Our study provides a new mechanistic understanding of how Aβ, particularly Aβ42, induces translational suppression, a process known to be exaggerated and to contribute to neurodegeneration in AD (Beckelman et al., 2019; Ma et al., 2013; Yang et al., 2016). Specifically, we showed that Aβ42 induces the expression of *Fmr1*, which represses the expression of PP1 and PP2A, leading to phosphorylation of eIF2α and eEF2 and subsequent translational suppression (Figure 8). These findings indicate that both PP2A and PP1 could be putative targets of therapeutic intervention in AD, which has also been proposed by others (Braithwaite et al., 2012; Torrent & Ferrer, 2012). However, there remain several questions that we hope to address in the future.

First, *Fmr1* is known to participate in many signaling pathways associated with translational suppression and neural activity homeostasis even at the basal state. Although our focus is on acute Aβ42‐induced changes, we cannot rule out the possibility that the chronic deficiency of FMRP in *Fmr1* KO mice changes the way that a neuron responds to challenges through a compensatory mechanism. A valuable future direction will be to employ shRNA against *Fmr1* to evaluate the effects following acute knockdown of *Fmr1* in Aβ42‐induced cellular stress signaling. This acute knockdown of *Fmr1* could also help ease the concern of potential genetic defects resulting from inbreeding of mouse colonies, which is a limitation of our current study. Also, while Aβ42‐induced translational suppression has been extensively observed in the past (Beckelman et al., 2019; Ding et al., 2005; Hernández‐Ortega et al., 2016; Langstrom et al., 1989; Oliveira et al., 2021; Radford et al., 2015; Sajdel‐Sulkowska & Marotta, 1984), a recent study has shown a role of FMRP in promoting translation in Aβ pathology (Ghosh et al., 2020). This discrepancy is likely caused by different experimental systems employed in different studies, but it also emphasizes the complexity of AD and the effort needed for its research.

Second, despite our discovery of *Fmr1*‐ and PP2A‐dependent hyposynchrony of neural network activity induced by Aβ42, the underlying mechanism remains unclear. Our current study suggests that the reduced number of excitatory synapses is unlikely to be one of the contributing factors (Figure 5). Another plausible mechanism to explain Aβ42‐induced hyposynchrony would be altered GABAergic inhibitory transmission. Extensive studies have illustrated an altered release of GABA or the impaired modulation of GABA receptors in AD (Jiménez‐Balado & Eich, 2021). Although the majority of studies have observed reduced GABAergic signal and hypersynchrony in animal models of severe or late‐stage AD (Xu et al., 2020), multiple studies have instead described elevated GABAergic signals during the early progression of AD (Hollnagel et al., 2019; Tang et al., 2020), which is similar to the acute model of Aβ pathology employed in our current study. Therefore, activation of the GABAergic system following Aβ treatment may be responsible for the hyposynchrony of neural activity. While our model of cultured neurons allows us to dissect out molecular mechanisms, it has limited capability to analyze effects at the circuit level or under a chronic accumulation of Aβ. To study GABAergic signaling especially following a chronic accumulation of Aβ, we will require in vivo or ex vivo preparations and could indicate a future research direction.

Third, the expression of FMRP in Aβ pathology remains controversial since two previous studies that evaluated the expression of FMRP in mouse models of Aβ pathology observed different results: one study observed an elevation of FMRP (Hamilton et al., 2014) whereas the other one did not (Renoux et al., 2014). We speculate that this discrepancy might be related to the levels of APP or Aβ in the mice that were used in different studies. This prediction is based on a previous study showing the role of FMRP in repressing the expression of APP (Westmark et al., 2016). Elevation of FMRP following an acute or initial encounter with Aβ may lead to a reduction of APP. A reduction of APP theoretically would lead to a reduction of Aβ, subsequently diminishing the effect on the elevation of FMRP. This feedback loop needs further validation but could certainly explain an insignificant elevation of FMRP in an AD animal model that was reported previously (Renoux et al., 2014).

Fourth, although the exaggerated translational suppression was well documented in AD in vitro and in vivo, the underlying mechanisms are complicated and not fully understood. In addition to global translational suppression through eIF2α as previously discovered (Ma et al., 2013) and through eEF2 as we have described in our current study, elevated translational suppression has also been shown to be correlated to enhanced formation of stress granules (Wolozin & Ivanov, 2019). Stress granules are a cytosolic aggregation of translational machinery, RNA‐binding proteins, and RNAs that function to repress translation under stress. In AD, stress granules are abnormally promoted (Sidibé & Vande Velde, 2019; Wolozin & Ivanov, 2019), which further facilitates translational suppression. Because FMRP is also a key constituent of stress granules (Lai et al., 2020; Valdez‐Sinon et al., 2020) and both PP2A and PP1 can indirectly regulate stress granule dynamics (Kedersha et al., 2013; Shelkovnikova et al., 2017), it would be particularly interesting to know whether stress granules can be elevated in our acute model of Aβ pathology and, if yes, whether FMRP is involved in the process.

Lastly, while our study focuses on neurons when we measured neural network activity and synapse numbers, all the critical components in our research are known to be expressed in glial cells as well. The involvement of glial cells in AD has been well established (Ries & Sastre, 2016; Ziegler‐Waldkirch & Meyer‐Luehmann, 2018), and cellular stress‐induced translational suppression has been reported in glial cells (Wang et al., 2010). Whether, and to what extent Aβ triggers similar translational suppression in glial cells is still unclear. Assuming that exaggerated translational suppression is harmful to the cells following treatment with Aβ, glial cells may also be affected by the same phenomenon, leading to neurodegeneration. As a future direction, we hope to characterize the cell‐type specificity of our pathway (Figure 7), particularly in glial cells. Ultimately, we hope that our long‐term efforts will provide comprehensive mechanistic insights surrounding translational suppression in the understanding and treatment of AD.

## CONFLICTS OF INTEREST

The authors declare no conflicts of interest.

## AUTHOR CONTRIBUTIONS

Simon Lizarazo and Nien‐Pei Tsai designed the research. Simon Lizarazo and Yeeun Yook performed the research and analyzed the data. Simon Lizarazo and Nien‐Pei Tsai wrote the manuscript. All authors read and approved the final manuscript.

## Supporting information

Supporting information.
