# Supplementary material for: Amyloid beta induces Fmr1‐dependent translational suppression and hyposynchrony of neural activity via phosphorylation of eIF2α and eEF2
Source: J Cell Physiol. 2022 Apr 17;237(7):2929–42. doi: 10.1002/jcp.30754 (PMC9283232; doi:10.1002/jcp.30754)
Supplement: Supplementary file 1 — Supporting information. [file JCP-237-2929-s001.pdf]

Amyloid beta induces *Fmr1*-dependent translational suppression and hyposynchrony of neural activity via phosphorylation of eIF2α and eEF2

Simon Lizarazo[1], Yeeun Yook[1] and Nien-Pei Tsai[1,2,*]

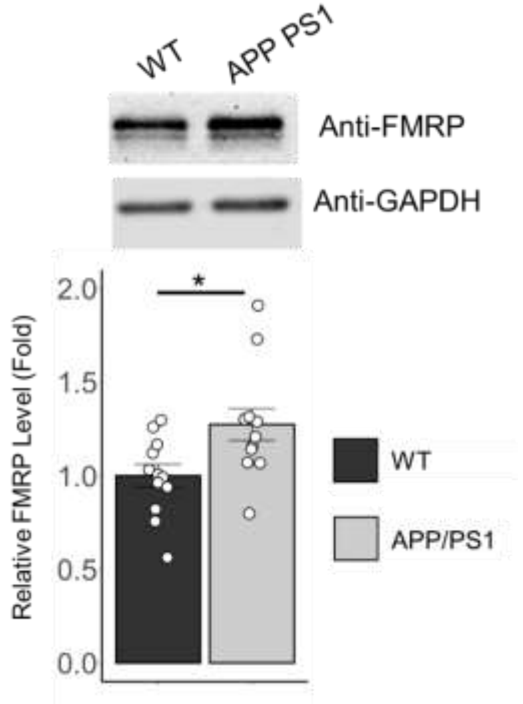

Supplementary Figure 1. FMRP is elevated in APP/PS1 mice. Representative western blot and quantification of FMRP and GAPDH from WT and APP PS1 brain lysates obtained from 12 weeks old mice (n = 12 and 11 for WT and APP/PS1, respectively). No data points were removed after the Grubbs' outlier test. Student's *t*-test was used. Data are represented as mean ± SEM with $^*P<0.05$.

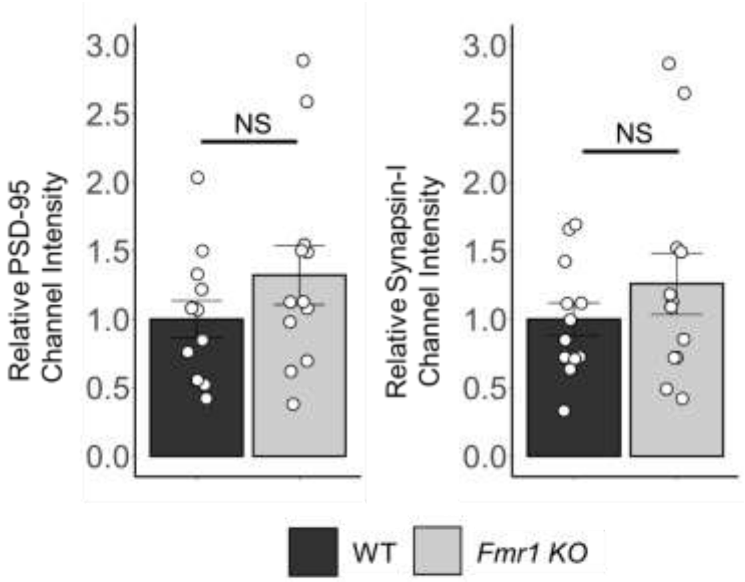

Supplementary Figure 2. PSD-95 and Synaptic-I are similarly expressed in WT and *Fmr1* KO neuron at the basal state. Basal channel intensity for post-synaptic marker (PSD-95) and pre-synaptic marker (Synapsin-I). Quantification was made using imageJ software measuring the basal channel intensity of secondary dendrites from WT and *Fmr1* KO cortical neurons. Normalization was made related to basal intensity of the dendritic marker (MAP2). Data were collected from two independent cultures with n = 6 cells from each culture for both WT and *Fmr1* KO. No data points were removed after the Grubbs' outlier test. Student's t-test was used. Data are represented as mean ± SEM with ns: non-significant.

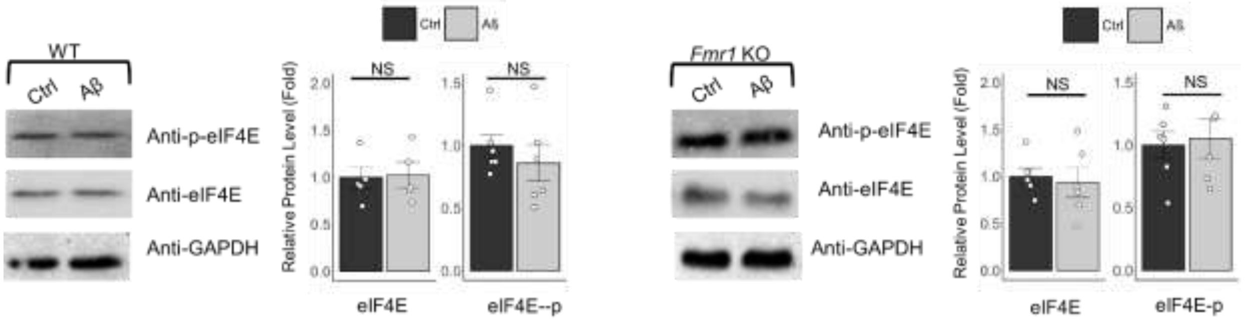

Supplementary Figure 3. Treatment of Aβ does not alter the expression level and phosphorylation status of eIF4E. Representative western blots and quantifications of eIF4E, p-eIF4E and GAPDH from WT (left) and *Fmr1* KO (right) cortical neuron cultures treated with amyloid beta 1-42 (Aβ; 1 μM) or scrambled Aβ peptide (Ctrl, 1 μM) for 24 hours at DIV 12-14. (n = 5-6 from 3 independent cultures after removing one WT culture treated with scrambled peptide for analysis of eIF4E following the Grubbs' outlier test.). Student's t-test was used. Data are represented as mean ± SEM with ns: non-significant.

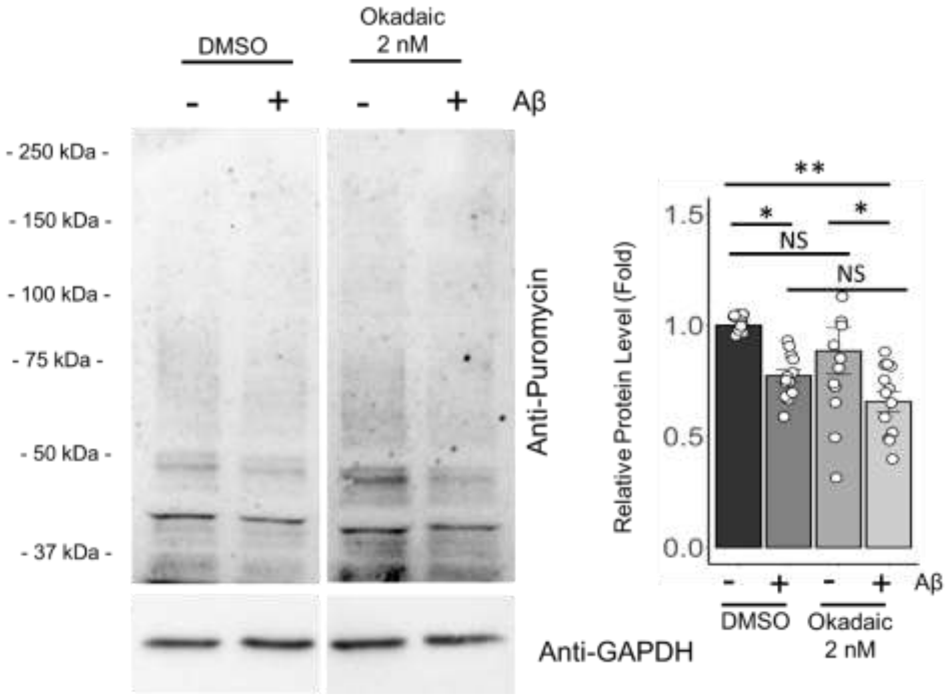

Supplementary Figure 4. Inhibition of PP2A exerts slight but not significant effects on translational suppression in WT neurons. Representative western blots and quantifications of puromycin and GAPDH from WT cortical neuron cultures treated with amyloid beta 1-42 (Aβ; 1 μM) or scrambled Aβ peptide (Ctrl, 1 μM) for 24 hours and with vehicle (DMSO) or okadaic acid (2 nM or 100 nM) for the last hour at DIV 12-14. (n = 12 independent cultures after removing one culture treated with scrambled peptide + okadaic acid following the Grubbs' outlier test.). Two-way ANOVA with Tukey test were used (Interaction: $F_{1,47}$=0.000, p=0.991; peptide effect: $F_{1,47}$=15.802, p=0.0002; drug effect: $F_{1,47}$=3.999, p=0.051). Data are represented as mean ± SEM with *P<0.05, **P<0.01, ns: non-significant.

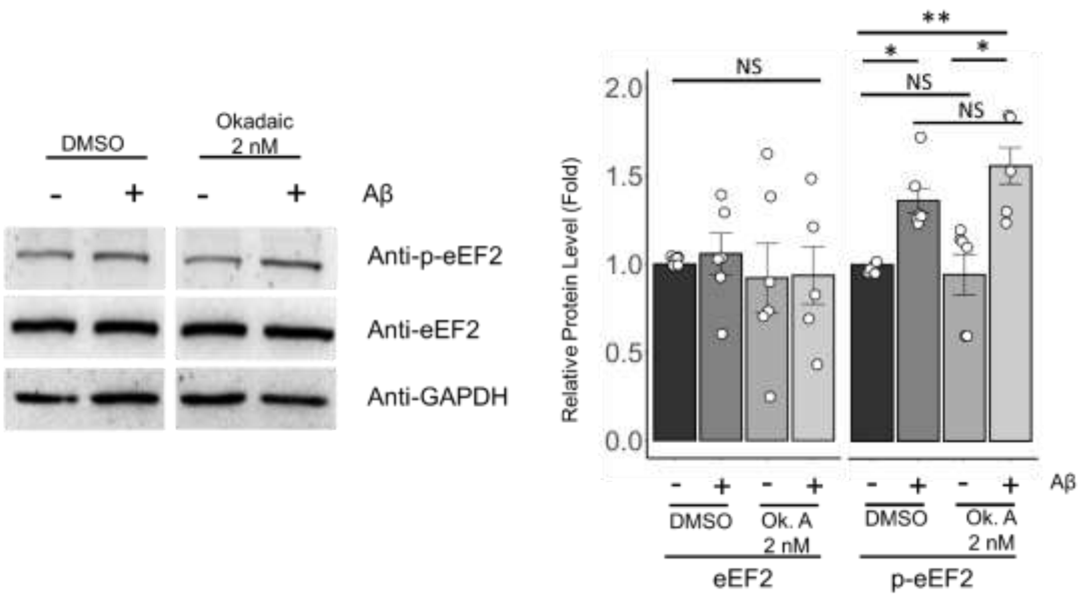

Supplementary Figure 5. Inhibition of PP2A does not exert significant further effects on eEF2 phosphorylation in WT neurons. Representative western blots and quantifications of eEF2, p-eEF2 and GAPDH from WT cortical neuron cultures treated with amyloid beta 1-42 (Aβ; 1 μM) or scrambled Aβ peptide (Ctrl, 1 μM) for 24 hours and with vehicle (DMSO) or okadaic acid (2 nM or 100 nM) for the last hour at DIV 12-14. (n = 5-6 independent cultures after removing one culture treated with amyloid beta + okadaic acid for analysis of eEF2 and p-eEF2 following the Grubbs' outlier test.) Two-way ANOVA with Tukey test were used (For the left panel, interaction: $F_{1,19}=0.024$, $p=0.879$; peptide effect: $F_{1,19}=0.086$, $p=0.772$; drug effect: $F_{1,19}=0.490$, $p=0.492$. For the right panel, interaction: $F_{1,19}=2.141$, $p=0.160$; peptide effect: $F_{1,19}=31.342$, $p=0.00002$; drug effect: $F_{1,19}=0.566$, $p=0.461$). Data are represented as mean ± SEM with *P<0.05, **P<0.01, ns: non-significant.
